# Watermelon Duodenum (Duodenal Bulb Vascular Ectasia) in a Patient With Hepatocellular Carcinoma: A Rare Cause of Upper Gastrointestinal Bleeding

**DOI:** 10.7759/cureus.101211

**Published:** 2026-01-10

**Authors:** Abdullah Alhouri, Sara Alhajjar, Muhammad U Khalid, Faisal Nawaz

**Affiliations:** 1 Gastroenterology, Aneurin Bevan University Health Board, Wales, GBR; 2 Pharmacology, Al-Sham Private University, Damascus, SYR; 3 Gastroenterology, Isle of Wight NHS Trust, Newport, GBR; 4 Gastroenterology and Hepatology, The Grange University Hospital, Cwmbran, GBR

**Keywords:** duodenal bulb vascular ectasia, gastric antral vascular ectasia (gave), gastrointestinal bleeding, hepatic encephalopathy, watermelon duodenum

## Abstract

Watermelon duodenum, also known as duodenal bulb vascular ectasia (DBVE), is a rare and under-recognized cause of upper gastrointestinal bleeding. Unlike its more commonly reported gastric counterpart - gastric antral vascular ectasia (GAVE) - DBVE is seldom described in the literature, and its clinical presentation, risk factors, and optimal management remain poorly defined.

We report the case of an elderly patient with multiple complex comorbidities, including hepatocellular carcinoma, atrial fibrillation on anticoagulation, and chronic immune thrombocytopenia, who presented with worsening confusion and lethargy consistent with hepatic encephalopathy. Laboratory studies revealed anemia and elevated ammonia levels, raising suspicion for an upper gastrointestinal source of bleeding as a precipitating factor. Urgent esophagogastroduodenoscopy demonstrated isolated, longitudinally arranged vascular ectatic lesions confined to the duodenal bulb, producing a characteristic “watermelon” appearance. No involvement of the gastric antrum was noted. The lesions were treated endoscopically with hemostatic therapy, argon plasma coagulation (APC), resulting in clinical stabilization and improvement of encephalopathy.

This case emphasizes the need for heightened clinical awareness of DBVE as a potential source of upper gastrointestinal bleeding. Early identification and appropriate endoscopic management can significantly improve outcomes, particularly in patients with complex comorbid profiles.

## Introduction

Gastric antral vascular ectasia (GAVE), also known as “watermelon stomach,” is a well-described but relatively uncommon cause of chronic gastrointestinal bleeding [1]. It accounts for up to 4% of non-variceal upper gastrointestinal hemorrhage and typically presents with iron deficiency anemia, occult bleeding, or recurrent melena [1]. Histologically, GAVE is characterized by mucosal vascular ectasia, fibrin thrombi within capillaries, and fibromuscular hyperplasia of the lamina propria [1]. The classical endoscopic appearance is that of longitudinal red streaks radiating outward from the pylorus, resembling the stripes of a watermelon [1].

GAVE is frequently associated with systemic conditions, particularly cirrhosis and portal hypertension, but also with autoimmune diseases (such as systemic sclerosis and CREST syndrome), renal insufficiency, and cardiovascular disorders [2]. Interestingly, despite the strong association with cirrhosis, GAVE is thought to arise from mechanisms beyond portal hypertension, as it typically responds poorly to interventions that reduce portal pressure (e.g., β-blockade and transjugular intrahepatic portosystemic shunt (TIPS)) [2-4].

In contrast, duodenal bulb vascular ectasia (DBVE), or “watermelon duodenum,” is an exceedingly rare entity, with only sporadic case reports in the literature [4-6]. This condition is believed to share pathophysiological mechanisms with GAVE, including mechanical stress from abnormal peristalsis, mucosal prolapse, and vascular injury leading to ectasia [4]. Clinically, it may manifest as occult bleeding, melena, or decompensation of chronic liver disease due to anemia or hepatic encephalopathy [4]. Given its rarity, diagnosis is often delayed, and its management is primarily derived from that of GAVE.

This report discusses a 72-year-old male patient with hepatocellular carcinoma and multiple comorbidities who experienced hepatic encephalopathy secondary to upper gastrointestinal bleeding from watermelon duodenum, emphasizing the diagnostic challenge and therapeutic considerations.

## Case presentation

A 72-year-old male was admitted with acute confusion and melena. His past medical history included immune thrombocytopenia, mild chronic obstructive pulmonary disease (ex-smoker, quit 30 years ago), chronic kidney disease stage 3, atrial fibrillation on apixaban (with two previous cardioversions), hepatocellular carcinoma (treated with chemoembolization in December 2024 and five cycles of radiotherapy completed in May 2025), heart failure with preserved ejection fraction, and hypertension. On examination, he was hemodynamically stable but confused. The abdomen was soft and non-tender; the chest was clear; and heart sounds were normal, with an irregularly irregular rhythm consistent with atrial fibrillation. Digital rectal examination revealed black, tarry stool. Laboratory investigations revealed severe anemia (hemoglobin 66 g/L), leukopenia (white cell count 3.5 × 10⁹/L), thrombocytopenia (platelets 81 × 10⁹/L), and chronic renal impairment, with elevated creatinine (191 µmol/L), elevated urea (16 mmol/L), ammonia level 117 µmol/L, and a reduced estimated glomerular filtration rate (eGFR 30 mL/min). The remaining biochemical, coagulation, and liver function tests were unremarkable (Table 1). The patient had a Child-Pugh score of 6 (Class A), consistent with compensated cirrhosis. The etiology of his liver disease was hepatocellular carcinoma-related cirrhosis, and imaging demonstrated no evidence of portal vein thrombosis.

**Table 1:** Summary of the laboratory results Laboratory values for the patient - including hematology, renal function, liver function, coagulation, and inflammatory markers - are presented. Patient results are shown alongside standard reference ranges.

Blood Test	Patient Value	Reference Range
Hemoglobin (Hb)	66 g/L	115-160 g/L
White Cell Count (WCC)	3.5 × 10⁹/L	4.0-11.0 × 10⁹/L
Platelet Count (Plt)	81 × 10⁹/L	150-400 × 10⁹/L
Creatinine (Cr)	191 µmol/L	45-90 µmol/L
Urea	16 mmol/L	2.5-7.8 mmol/L
Estimated Glomerular Filtration Rate (eGFR)	30 mL/min	>60 mL/min
Sodium (Na⁺)	142 mmol/L	135-145 mmol/L
Potassium (K⁺)	3.6 mmol/L	3.5-5.1 mmol/L
Prothrombin Time (PT)	12.4 seconds	9-12 seconds
Activated Partial Thromboplastin Time (aPTT)	31.4 seconds	25-35 seconds
Fibrinogen	2.6 g/L	1.5-4.5 g/L
Alanine Aminotransferase (ALT)	21 U/L	10-49 U/L
Alkaline Phosphatase (ALP)	91 U/L	30-130 U/L
Bilirubin	17 µmol/L	0-21 µmol/L
C-Reactive Protein (CRP)	<10 mg/L	<10 mg/L
Ammonia	117 µmol/L	<50 µmol/L

**Figure 1:**
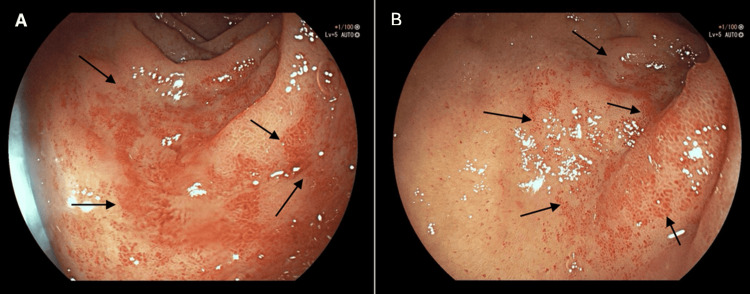
Endoscopic appearance of duodenal vascular ectasias (watermelon duodenum) Labels A and B are endoscopic images of the duodenal bulb, demonstrating extensive areas of mucosal involvement by multiple small vascular ectasias, which are indicated by the arrows in the images.

CT head was unremarkable. Upper gastrointestinal endoscopy demonstrated a sliding hiatal hernia and an unremarkable gastric examination without any significant lesions. However, the duodenal bulb revealed multiple small vascular ectasias consistent with “watermelon duodenum” (DBVE) (Figures 1A-1B). The second part of the duodenum appeared normal. The lesions were treated endoscopically with argon plasma coagulation (APC), resulting in clinical stabilization and improvement of melena and encephalopathy. The patient received two units of packed red blood cells, with an improvement in hemoglobin to 89 g/L. Carvedilol was commenced for portal hypertension prophylaxis, with monitoring for hypotension and bradycardia. Apixaban was safely reintroduced before discharge, with a plan to perform further APC of DBVE in case of any further hemoglobin drop or melena. A follow-up at three weeks showed no recurrence of bleeding.

## Discussion

This case underscores several essential aspects of watermelon duodenum as a rare but clinically significant cause of gastrointestinal bleeding [4]. Although better recognized in the stomach as GAVE, the occurrence of vascular ectasia in the duodenum remains extremely uncommon, with only a few cases reported in the literature [4].

The exact pathogenesis of DBVE remains uncertain, but several mechanisms have been proposed, most of which are derived from studies on GAVE [1,7]. Mechanical factors are thought to play a role, with abnormal peristalsis and mucosal prolapse leading to repetitive trauma of the duodenal mucosa [4,8]. This chronic insult may promote dilatation of mucosal vessels and fibromuscular hyperplasia [4,8]. In addition, systemic and vascular factors are implicated, as DBVE has been described in association with cirrhosis, renal failure, and cardiac disease, suggesting that vascular dysregulation and impaired mucosal perfusion may contribute to its development [4]. An autoimmune component has also been considered, since case series have reported an association between vascular ectasias and connective tissue disorders, raising the possibility of immune-mediated endothelial injury [9]. Interestingly, while portal hypertension is often present in patients with GAVE and DBVE, it is not believed to be the primary driver [9]. This is supported by the observation that lesions rarely regress following portal pressure-lowering interventions, such as TIPS or pharmacological therapy with beta-blockers [9].

Clinically, patients with DBVE may present with a range of manifestations, including iron-deficiency anemia, recurrent melena, or overt upper gastrointestinal bleeding [4]. In cases of cirrhosis or hepatocellular carcinoma, bleeding may trigger hepatic encephalopathy, as was observed in our patient [9]. The endoscopic appearance of DBVE is distinctive, with longitudinal erythematous streaks resembling watermelon stripes in the duodenal bulb. However, recognition may be challenging due to overlap with other vascular lesions [6]. The main differential diagnoses consist of duodenal angiodysplasia, characterized by punctate lesions instead of linear streaks; portal hypertensive gastropathy or duodenopathy, which manifests as a mosaic-like pattern; and hereditary hemorrhagic telangiectasia or other telangiectatic syndromes [10,11]. Careful endoscopic evaluation and awareness of the unique streak-like pattern are therefore essential in distinguishing DBVE from these mimics.

Management of watermelon duodenum is primarily extrapolated from the experience with GAVE [12]. Endoscopic therapy is generally considered the mainstay of treatment in symptomatic patients [12]. APC has been the most widely used technique, demonstrating high efficacy and low complication rates [12]. Other modalities, such as heater probe coagulation, radiofrequency ablation, and endoscopic band ligation, have also been employed in selected cases [7,12]. Surgical interventions, including antrectomy or duodenectomy, are infrequently warranted due to their significant morbidity and are generally reserved for refractory or uncontrolled cases [7].

Supportive measures remain critical in the overall management strategy. These include blood transfusions for acute anemia, iron supplementation for chronic losses, and optimization of underlying comorbidities, such as cirrhosis, renal dysfunction, and cardiac disease. In our patient, given the burden of comorbidities and the absence of active hemorrhage, a conservative strategy was adopted. He was managed with transfusion support and initiation of carvedilol for portal hypertensive prophylaxis, along with close monitoring. This tailored approach highlights the need to balance the risks and benefits of invasive therapy in medically complex patients.

## Conclusions

Watermelon duodenum is a rare cause of upper gastrointestinal bleeding that can present with life-threatening complications, especially in patients with advanced liver disease. Awareness of this condition is essential, as recognition allows for targeted endoscopic intervention or conservative management depending on patient factors. Reporting such cases adds to the limited literature and may help guide future diagnostic and therapeutic strategies.
